# Turbulent Kinetic Energy Measurement Using Phase Contrast MRI for Estimating the Post-Stenotic Pressure Drop: *In Vitro* Validation and Clinical Application

**DOI:** 10.1371/journal.pone.0151540

**Published:** 2016-03-15

**Authors:** Hojin Ha, Guk Bae Kim, Jihoon Kweon, Hyung Kyu Huh, Sang Joon Lee, Hyun Jung Koo, Joon-Won Kang, Tae-Hwan Lim, Dae-Hee Kim, Young-Hak Kim, Namkug Kim, Dong Hyun Yang

**Affiliations:** 1 POSTECH Biotech Center, Pohang University of Science and Technology, Pohang, South Korea; 2 Asan Institute of Life Science, Asan Medical Center, University of Ulsan College of Medicine, Seoul, South Korea; 3 Department of Cardiology, University of Ulsan College of Medicine, Asan Medical Center, Seoul, South Korea; 4 Department of Mechanical Engineering, Pohang University of Science and Technology, Pohang, South Korea; 5 Department of Radiology, University of Ulsan College of Medicine, Asan Medical Center, Seoul, South Korea; 6 Department of Convergence Medicine, University of Ulsan College of Medicine, Asan Medical Center, Seoul, South Korea; University of California San Diego, UNITED STATES

## Abstract

**Background:**

Although the measurement of turbulence kinetic energy (TKE) by using magnetic resonance imaging (MRI) has been introduced as an alternative index for quantifying energy loss through the cardiac valve, experimental verification and clinical application of this parameter are still required.

**Objectives:**

The goal of this study is to verify MRI measurements of TKE by using a phantom stenosis with particle image velocimetry (PIV) as the reference standard. In addition, the feasibility of measuring TKE with MRI is explored.

**Methods:**

MRI measurements of TKE through a phantom stenosis was performed by using clinical 3T MRI scanner. The MRI measurements were verified experimentally by using PIV as the reference standard. *In vivo* application of MRI-driven TKE was explored in seven patients with aortic valve disease and one healthy volunteer. Transvalvular gradients measured by MRI and echocardiography were compared.

**Results:**

MRI and PIV measurements of TKE are consistent for turbulent flow (0.666 < R^2^ < 0.738) with a mean difference of −11.13 J/m^3^ (SD = 4.34 J/m^3^). Results of MRI and PIV measurements differ by 2.76 ± 0.82 cm/s (velocity) and −11.13 ± 4.34 J/m^3^ (TKE) for turbulent flow (Re > 400). The turbulence pressure drop correlates strongly with total TKE (R^2^ = 0.986). However, *in vivo* measurements of TKE are not consistent with the transvalvular pressure gradient estimated by echocardiography.

**Conclusions:**

These results suggest that TKE measurement via MRI may provide a potential benefit as an energy-loss index to characterize blood flow through the aortic valve. However, further clinical studies are necessary to reach definitive conclusions regarding this technique.

## Introduction

The transvalvular pressure gradient (TPG) is an important parameter that characterizes the hemodynamic performance of heart valves. To measure the pressure change across a heart valve, the use of a pressure catheter has proven to be straightforward and accurate. Alternatively, TPG can be noninvasively estimated by using Doppler echocardiography, which measures blood velocity at the valve level and estimates the pressure gradient by using a simplified Bernoulli equation [1]. However, discrepancies often appear between catheter- and Doppler-based pressure measurements, mostly because echocardiography overestimates TPG by not accounting for the pressure recovery [2]. Recently, the quantification of turbulence kinetic energy (TKE) using four-dimensional (4D) phase-contrast magnetic resonance imaging (PC-MRI) was introduced as an alternative method for predicting the turbulence energy loss of the blood flow through the heart valve [3].

Previous work reported a method to measure energy loss in MRI by computing turbulence intensity and intravoxel standard deviation (IVSD) based on standard PC-MRI [4, 5]. Since quantifying TKE by using PC-MRI has been successful in several *in vitro* phantom studies [4–7], its clinical potential has drawn attention. A recent study reported that the total TKE in the ascending aorta of patients with aortic stenosis correlates with the conventional, Doppler-based pressure-loss index [3]. Despite the various studies that use MRI to quantify turbulence, several steps remain before this method can be employed in clinical practice. First, notwithstanding the numerous studies involving simulations [5, 8–10] and the experimental verification based on particle tracking velocimetry (PIV) [11], the accuracy of MRI-based TKE measurement over a range of turbulent-flow rates is not yet fully verified, because few research groups have established the TKE quantification method and experimentally compared the TKE quantification with a conventional, gold-standard method. That said, various measurement conditions such as the geometry of the vessel, flow conditions, and even sequences from the different vendors are known to influence quantification of TKE. Second, although the in vivo feasibility of TKE quantification has been investigated for cardiovascular flow, such as the aortic stenosis, aortic coarctation, and mitral regurgitation [3, 12, 13], the clinical feasibility of TKE measurement by using 4D PC-MRI for various valvular diseases has received little attention. Therefore, the primary goal of this study is to verify the 4D PC-MRI measurement of the velocity and TKE through a phantom stenosis with PIV serving as a comparator. In addition, the feasibility of using 4D PC-MRI to measure TKE in the clinic is investigated in a group of patients with aortic valve disease.

## Methods

### Stenosis-Phantom Fabrication

By using a three-dimensional (3D) printer (Fortus 400mc, Stratasys), a stenosis model was fabricated based on a cosine-form formula with 50% and 75% reduction in the diameter and cross-sectional area, respectively [14, 15]. The diameter (D) was 25 mm and the total length of the model was 22 D or 550 mm. The lengths upstream and downstream of the stenosis were 10 D. The length of the stenotic region was 2 D. The printed stenosis model was cast from polydimethylsiloxane (PDMS, Sylgard 184, Dow Corning, USA).

### Flow Circuit System and Pressure Measurement

S1a Fig schematizes the flow-circulating system used in this study. The working fluid (9 L), prepared in an acrylic reservoir, consisted of a 60:40 glycerol/water solution. The density and dynamic viscosity of the working fluid were 1148 kg/m^3^ and 8.98 × 10^−3^ kg·m^−1^·s^−1^, respectively. The working fluid was circulated through the flow-circuit system at a constant flow rate by a centrifugal pump (Eheim Compact Plus 5000, Eheim, Germany). The flow rate was controlled by a flow valve and monitored by an electromagnetic flowmeter (VN10, Wintech Process, South Korea). The flow rate was varied from 1.3 to 8.7 L/min, which corresponds to Reynolds numbers (Re) from 114 to 945. In the present study, Re is estimated on the basis of the nonconstricted flow by the ratio QD/νA, where Q is the flow rate, D is the nonconstricted diameter of the phantom, ν is the kinematic viscosity, and A is the cross-sectional area of the channel. For pressure measurements, 2-mm-diameter pressure taps were made in the pre- and post-stenosis regions. The pressure drop across the stenosis was measured by using a U-tube manometer. For laminar flow, the pressure drop is linearly proportional to the flow rate, and the relationship between the pressure and the flow rate deviates from linearity as turbulence increases [16]. Therefore, the effect of turbulent flow on pressure drop is estimated by subtracting the laminar-flow pressure drop from the total pressure drop to correlate the TKE obtained by using 4D PC-MRI. In addition, the calculation of the turbulence pressure drop also cancels the influence of the pressure on the flow at the pre-stenosis and post-stenosis zones, where the flow is laminar. Therefore, the pressure drop may prove to be a more appropriate parameter than the measured pressure for investigating the relationship between pressure and TKE

### Imaging and Flow Parameters for *In Vitro* Phantom Studies

4D PC-MRI measurements were performed by using a clinical 3.0 T MRI scanner (Magnetom Skyra, Siemens, Germany). A gradient echo sequence with four-point velocity encoding was used. Velocity-encoding (VENC) parameters for measuring the velocity field were varied depending on the flow-rate conditions (see S1 Table). In comparison with the velocity measurements, smaller VENC parameters were used for TKE measurements to optimize the sensitivity of the method [5]. Echo time (TE) and flip angle were fixed at 6.76 ms and 10°, respectively. The temporal resolution was 92.72 ms and, for twofold acceleration along the phase-encoding direction, a partial Fourier acquisition (a factor of 6/8 along the phase- and frequency-encoding directions) and the integrated parallel acquisition technique (iPAT) with 32 reference lines were used. The matrix size was 256 × 256 × 6 with a voxel size of 1.4 mm × 1.4 mm × 1.4 mm. The minimum slice number was used to confine the region of interest for scanning at the center plane of the stenosis channel. The total scan time for each flow condition was about 100 s.

### Imaging and Flow Parameters for *In Vivo* Studies

Seven patients with valvular heart disease and one healthy volunteer were enrolled in this study. The institutional review board of the Asan Medical Center (Seoul, Korea) approved the study, and written informed consent was obtained from the patients. 4D PC-MRI measurements were made by using a clinical 3.0 T MRI scanner (Magnetom Skyra, Siemens, Germany), and a gradient echo sequence with four-point velocity encoding was used. VENC parameters for velocity-field measurements were determined by measuring the maximum velocity in the ascending aorta from two-dimensional (2D) PC-MRI. Compared with the velocity measurement, smaller VENC parameters were used for TKE measurements to optimize the sensitivity [5]. In particular, considering that the turbulence of the flow usually depends on the stenotic peak velocity, the VENC parameters for the in vivo TKE quantification required adjustment. Based on *in vitro* and *in vivo* preliminary studies, VENC parameters of about one-third of the peak velocity at the stenotic region resulted in relatively good sensitivity for quantifying the TKE. TE was set to the shortest possible value and the flip angle was fixed at 10°. The measurements used partial Fourier acquisition (a factor of 6/8 along the phase- and frequency-encoding directions) and the integrated parallel acquisition technique (iPAT) with 32 reference lines for twofold acceleration along the phase-encoding direction. The matrix size was 132 × 192 × (30–48) with a voxel size of 2.0 mm × 2.0 mm × 2.0 mm. In the present study, the electrocardiography gating was used to synchronize the data with the cardiac phase. However, respiratory gating was not used to reduce the scan time to compensate for the loss of effective spatial resolution. The total scan time was varied from 29 to 66 min depending on the scanning range and heart rate. Additional details are presented in S2 Table. Transthoracic echocardiography including Doppler imaging was done by using commercially available ultrasonographic equipment (Sonos 7500, Philips Medical Systems, Andover, USA) with a 3–5 MHz transducer. By using the blood-flow velocity measured at the valve level, the pressure drop was estimated from a simplified Bernoulli equation. To compensate for pressure recovery, the ascending aorta diameter was used as described earlier [17].

### Particle Image Velocimetry

S1b Fig illustrates the PIV system for measuring the velocity field. To illuminate the measurement plane, a 0.5-mm-thick laser sheet was generated by using a 1 W continuous diode-pumped solid-state laser (Shanghai Dream Lasers Technology Co., China). A high-speed camera with 1 k × 1 k pixel resolution (Fastcam SA1.1, Photron, USA) was used to capture flow images for velocity-field measurements. A camera was positioned perpendicular to the flow to measure velocity fields at the center plane of the stenosis. A high-speed camera captured 778 pairs of flow images at intervals of 1/1000 to 1/3000 s, depending on the flow rate. A total of 778 instantaneous vector fields were obtained and averaged to provide the mean velocity field of the flow with standard deviation.

PIV data were analyzed by using PIVview software (PIVview 2C, PIVTEC, Germany). A fast-Fourier-transform-based cross-correlation PIV algorithm was applied to the flow images acquired to extract instantaneous velocity fields. A multigrid interrogation-window scheme was adopted with interrogation windows of 64 × 64, 32 × 32, and 16 × 16 pixels with 50% overlap. The distance between two adjacent velocity vectors was 8 pixels, which corresponds to 0.29 mm. Since the spatial resolution of the PIV data is much less than that of the MRI data, the results were interpolated by averaging the eight nearest velocity data to match the spatial resolution of the MRI data. According to our previous studies, the precision of PIV for estimating the velocity under similar experimental conditions was about ±3% along the centerline and about ±40% near the wall [15, 18]. Further details on the principle and uncertainty of the PIV system have been described previously [14].

### Data Analysis and Estimating TKE

Velocity-field data of 4D PC-MRI were obtained from the online reconstruction of the MRI scanner by using a standard phase-difference algorithm (S2 Fig). To estimate TKE, raw k-space data were exported from the MRI scanner. An in-house script written in MATLAB (The MathWorks, Natick, USA) was used to reconstruct the magnitude images of four-point measurements (reference and three directional measurements) and obtain the TKE. The TKE per unit volume is estimated from σ as follows:

$$TKE=\frac{1}{2}\rho \sum_{i=1}^{3}\sigma_{i}^{2}\;[J\;m^{-3}]$$ (1)

where ρ is the fluid density and σ_i_ is the standard deviation in the i direction. Because the PIV measures only the flow field at the 2D center plane of the stenosis phantom, TKE was quantified at the same plane. In contrast, the total TKE for in vivo studies was estimated by integrating TKE over the entire thoracic aorta. To filter out the TKE of the thoracic aorta, the thoracic aorta was manually segmented by referring to the magnitude and phase data obtained from 4D PC-MRI.

To visualize the velocity field and quantify the TKE of *in vivo* cases, the peak systole phase of the data was selected by finding the temporal phase with the maximum flow rate, and the velocity field and TKE were analyzed at the systole phase.

The resulting velocity and TKE data were loaded and analyzed by using an in-house tool written in MATLAB and were visualized by using Tecplot 360 (Tecplot, Inc., Bellevue, USA). For further analysis, the spatial resolutions of velocity and TKE data obtained from 4D PC-MRI and PIV were registered by using Tecplot 360, and correlation and Bland–Altman analyses were conducted by using SigmaPlot 10.0 (Systat Software Inc., San Jose, USA).

### Estimation of Vorticity

One of the basic fluid-dynamic parameters describing the rotational motion of a fluid element is the vorticity vector of the flow. Vorticity ω in a 2D flow field is defined as the curl of the velocity vector $\vec{v}$:

$$\omega =\nabla \times \vec{v}=\frac{\partial v_{y}}{\partial x}-\frac{\partial v_{x}}{\partial y}$$ (2)

where **∇** is the del operator. In the present study, the vorticity map of the center-plane velocity field in the stenosis phantom was obtained to visualize the shear layers at the post-stenosis region.

## Results

### *In Vitro* Measurement of Velocity and TKE using 4D PC-MRI

**Fig 1:**
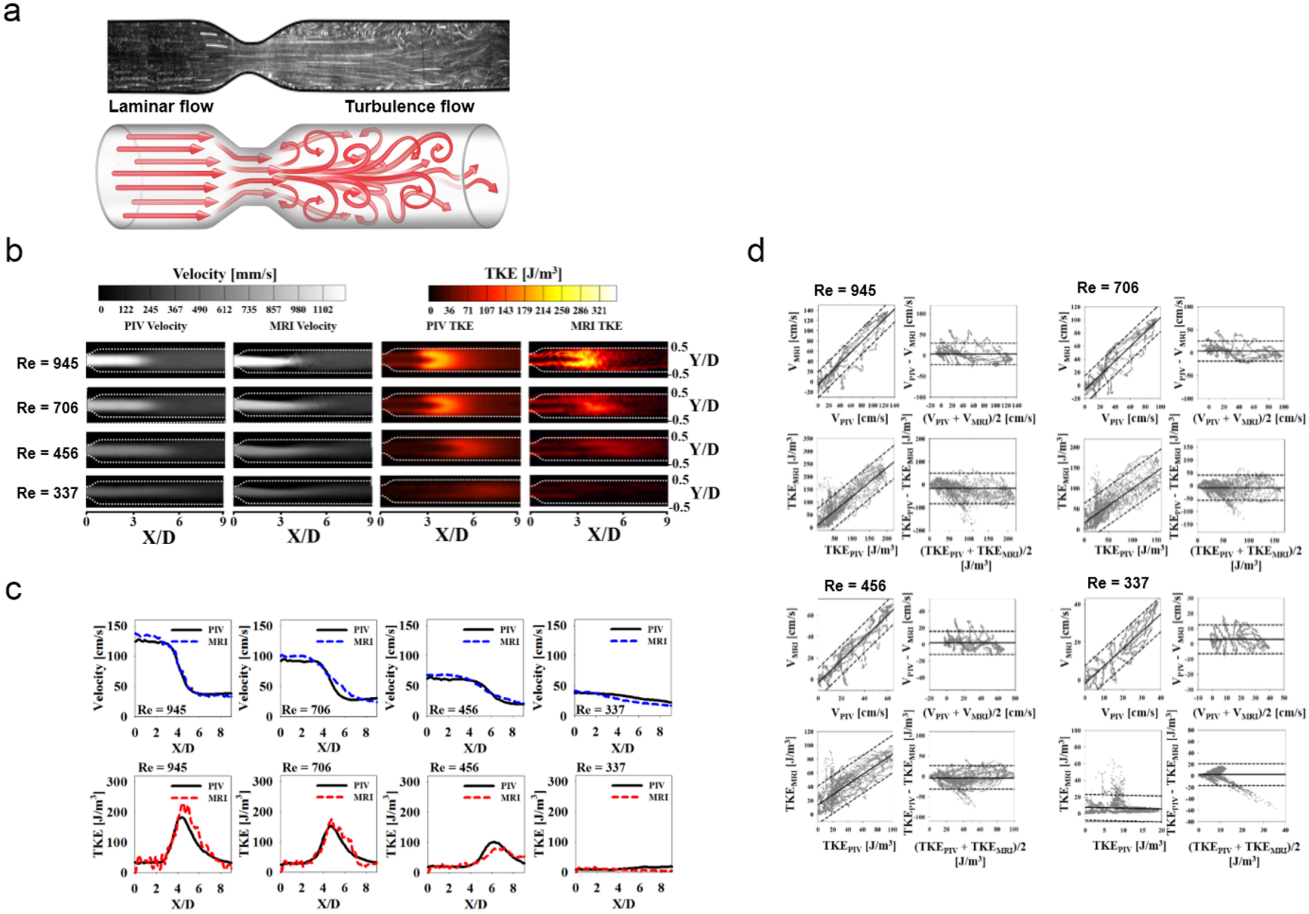
Comparison of 4D PC-MRI measurements using conventional PIV measurements. (a) Representative flow characteristics through a stenotic vessel. Flow visualization (upper panel) in PIV and schematic drawing (lower panel) indicate the development of a turbulent flow in the post-stenosis region. (b) Comparison of velocity and TKE distribution in the post-stenosis region. (c) Comparison of velocity (upper panel) and TKE (lower panel) along the centerline of the stenosis. (d) Linear regression and Bland–Altman analysis of velocity and TKE measured using PC-MRI and PIV at Re = 945, 706, 456, and 337. Note that solid and dotted lines in the linear regression indicate mean and 95% prediction band. Solid and dotted lines in the Bland–Altman plot indicate mean and 1.96 SDs.

Flow visualization shows that high turbulence occurs in the post-stenosis region (Fig 1a). Both measurements clearly show that the jet flow separates from the stenosis apex and dissipates as it travels downstream (Fig 1b). Locally high TKE distributions after the stenosis (3 < X/D < 6) are also detected by both measurements, where X/D is the axial length X from the stenosis apex normalized by the diameter D. Comparison of the velocity and TKE fields along the centerline of the stenosis shows that 4D PC-MRI provides the same outline as PIV (Fig 1c). Compared with the PIV measurement, 4D PC-MRI slightly overestimates the peak velocity at the stenosis apex by 9.63%, 7.80%, 8.53%, and 7.84% in flows with Reynolds numbers (Re = QD/νA) of 945, 706, 456, and 337, respectively.

Correlation and Bland–Altman analyses comparing 4D PC-MRI and PIV results for velocity and TKE are given in Fig 1d and summarized in S3 Table. The slope of the linear regression is closer to unity for the velocity measurements than for the TKE measurements. The 4D PC-MRI and PIV measurements differ by 2.76 ± 0.82 cm/s (velocity) and −11.13 ± 4.34 J/m^3^ (TKE) for turbulent flow (Re > 400).

**Fig 2:**
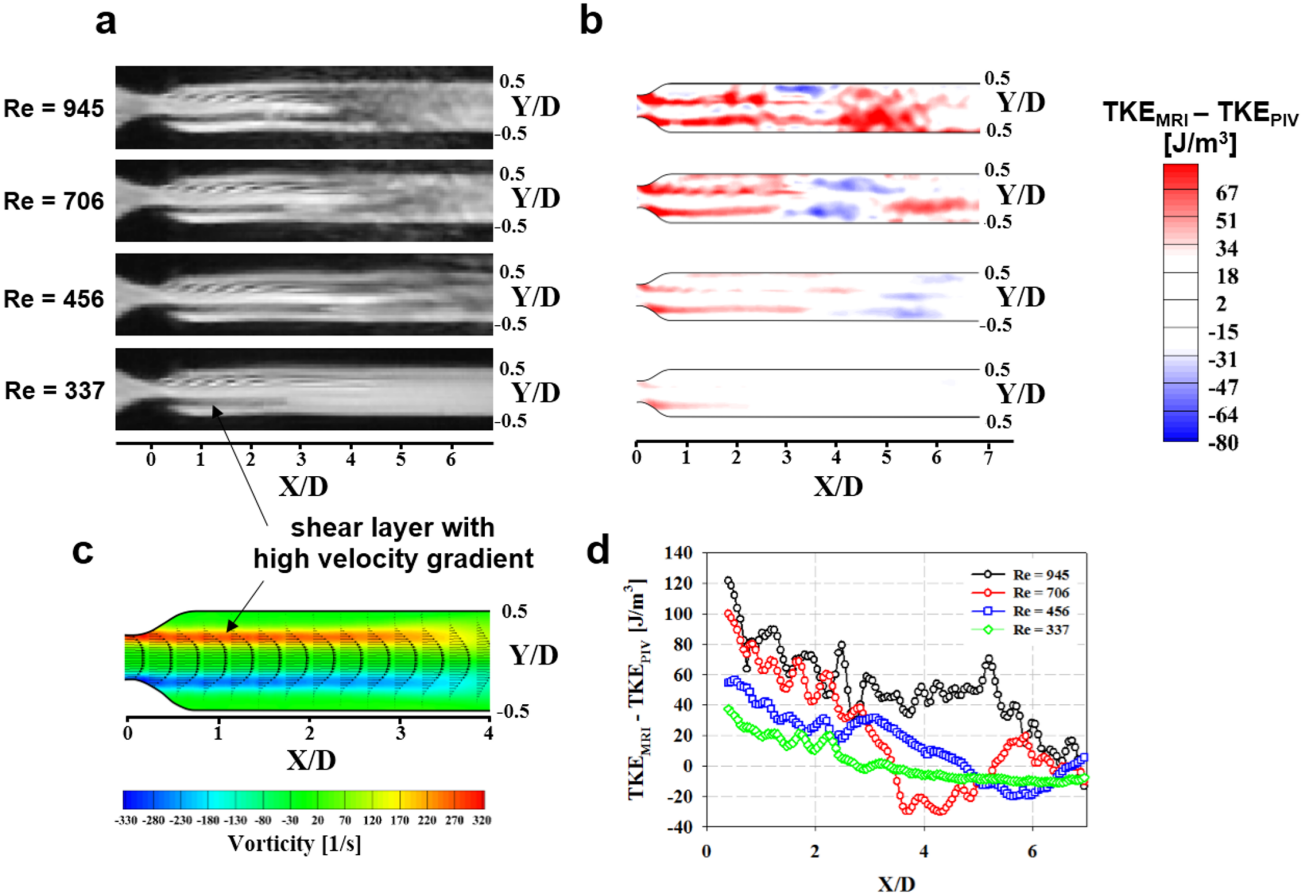
Overestimate of TKE_MRI_ at the shear-layer region. (a) Magnitude image. (b) Difference between TKE measured using MRI and that using PIV. (c) Velocity and vorticity map of flow in the post-stenosis region at Re = 945. (d) Variation in the TKE difference along the shear layer at Y/D = ±0.25. Each data point is the average of TKE differences at the shear layers at Y/D = −0.25 and 0.25. Note that the direction of the vorticity in panel (c) is out of the page.

The magnitude of the MRI-signal loss is excessively high in the post-stenosis region, which results in an overestimate of TKE by 4D PC-MRI compared with PIV (Fig 2a and 2b). The region where the TKE is overestimated coincides with the flow shear layer with a high spatial velocity gradient (Fig 2c). TKE is overestimated more severely upon approaching the stenosis (X/D < 2) than farther downstream (X/D > 2), regardless of Re (Fig 2d).

### Relationship between TKE and Post-Stenosis Pressure Drop

**Fig 3:**
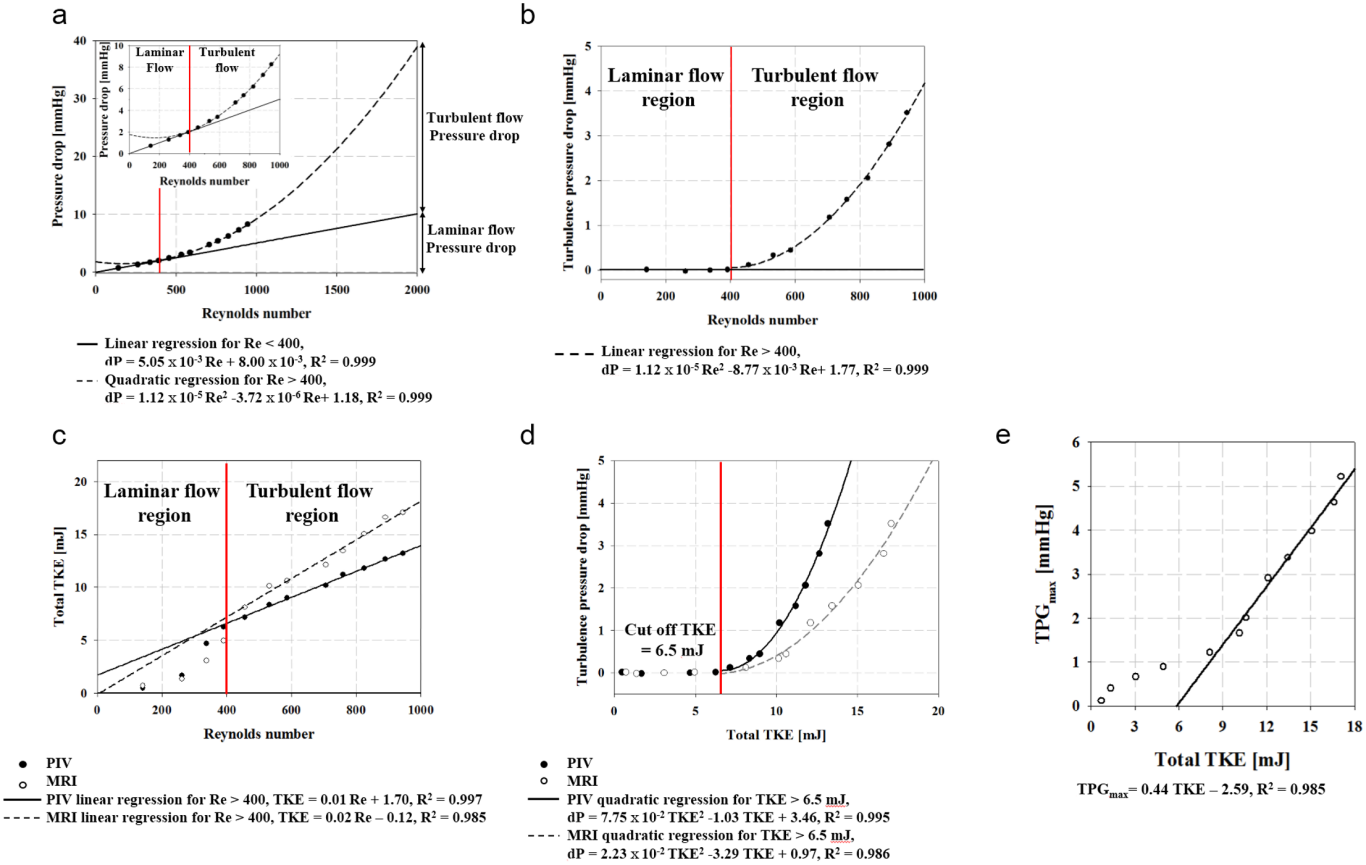
Relationship between TKE and pressure drop measured across stenosis phantom. (a) Pressure-drop measured through the stenosis. (b) Turbulence pressure drop across the stenosis channel. The pressure drop increases linearly for laminar flow (Re < 400) and quadratically for turbulent flow (Re > 400). (c) Total TKE variation at various values of Re. (d) Relationship between total TKE and turbulence pressure drop. (e) Relationship between TPG_max_ and total TKE.

Fig 3 shows the relationship between TKE and the pressure drop across the stenosis. The pressure drop (dP) increases linearly for laminar flow (Re < 400) and quadratically for turbulent flow (Re > 400). The regressions show that dP = 5.05 × 10^−3^ Re + 8.00 × 10^−3^ for laminar flow (R^2^ = 0.999) and dP = 1.12 × 10^−5^ Re^2^ − 3.72 × 10^−6^ Re + 1.18 for turbulent flow (R^2^ = 0.999). Turbulent pressure drop is estimated by subtracting the laminar-flow pressure drop from the total pressure drop, resulting in dP = 1.12 × 10^−5^ Re^2^ − 8.77 × 10^−3^ Re + 1.77 (R^2^ = 0.999).

The linear regression between total TKE and Re for turbulent flow is TKE = 0.01 Re + 1.70 for PIV (R^2^ = 0.997) and TKE = 0.02 Re − 0.12 for PC-MRI (R^2^ = 0.985). The discrepancy between the two measurements is due to an overestimate of TKE by PC-MRI. Consequently, the turbulence pressure drop is quadratically correlated with total TKE as follows: dP = 7.75 × 10^−2^ TKE^2^–1.03 TKE + 3.46 for PIV (R^2^ = 0.995) and dP = 2.23 × 10^−2^ TKE^2^ − 3.29 TKE + 0.97 for MRI (R^2^ = 0.986). The cutoff TKE for estimating the turbulence pressure drop is about 6.5 mJ. Total TKE and the Doppler-based maximum TPG (TPG_max_) are linearly correlated for turbulent-flow conditions where TKE exceeds the cutoff (Fig 3e). The linear regression results in TPG_max_ = 0.44 TKE − 2.59 (R^2^ = 0.985).

### *In Vivo* Application of 4D PC-MRI in Patients with Valvular Disease

**Fig 4:**
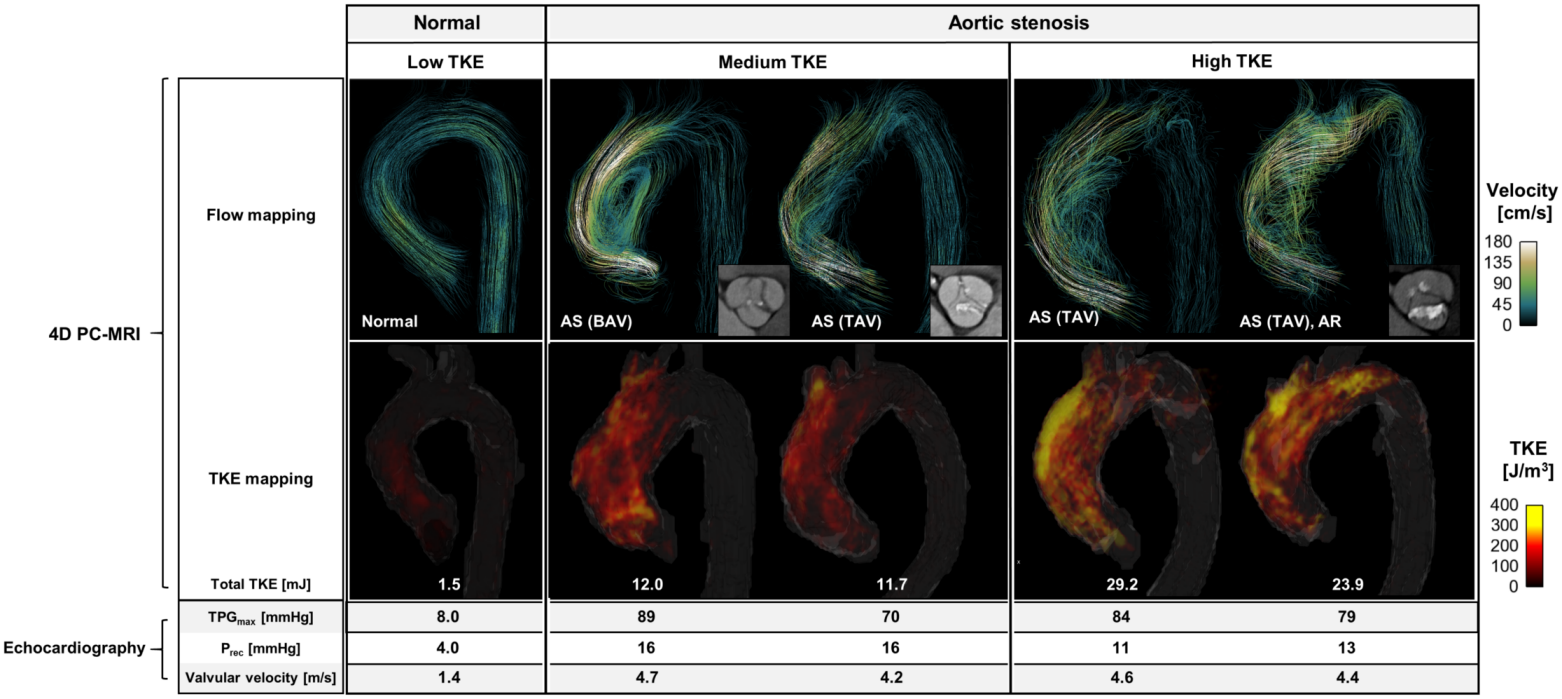
4D PC-MRI and echocardiographic findings in four patients with aortic stenosis. Flow mapping indicates the streamline of the flow. The uniform seed points within the thoracic aorta are used to visualize the streamlines. The TKE mapping is visualized by the volume rendering of the TKE within the entire thoracic aorta. AS: aortic stenosis, BAV: bicuspid aortic valve, TAV: tricuspid aortic valve, AR: aortic regurgitation, TPG_max_: maximum transvalvular pressure gradient, TKE: turbulence kinetic energy, and P_rec_: pressure recovery.

4D PC-MRI was applied to one healthy control patient and to seven patients with valvular disease (Table 1). Compared with the healthy control patient, the four patients with aortic stenosis had complex helical blood-flow patterns with higher flow velocities in the ascending aorta (Fig 4). TKE in the healthy control was low (<100 J/m^3^), whereas it was higher in the patients with aortic stenosis (>300 J/m^3^). When these patients were subdivided into two groups according to their total TKE values (two with medium TKE, <15 mJ; two with high TKE, >15 mJ), a discrepancy between TPG_max_ and total TKE is observed and the increase in TPG_max_ does not coincide with the increase in the TKE distribution.

**Table 1:** Demographic and clinical data of a healthy control patient and seven patients with valvular disease.

Variable	Case							
	# 1	# 2	# 3	# 4	# 5	# 6	# 7	# 8
Age, years	39	79	70	54	84	70	47	65
Sex	M	M	M	F	M	F	F	M
Disease	None	Aortic stenosis	Aortic stenosis, aortic regurgitation	Aortic stenosis	Aortic stenosis	Normal prosthetic valve	Abnormal prosthetic valve	Aortic regurgitation
Valve morphology	Tricuspid	Tricuspid	Tricuspid	Bicuspid	Tricuspid	Prosthetic	Prosthetic	Tricuspid
Aortic valve orifice area [mm^2^]	NA	NA	106.9	119.9	71.6	131.4	50.2	554.8
Total TKE [mJ]*	1.5	29.2	23.9	12.0	11.7	3.3	7.0	15.0 (7.7, diastole)
Peak velocity [m/s]	1.4	4.5	5.7	4.7	4.2	1.7	5.9	2.3
TPG_max_ [mmHg]	8	81	132	89	70	11	141	22
TPG_mean_ [mmHg]	5	51	79	54	42	6	100	7
BSA [m^2^]	1.9	1.8	1.9	1.5	1.8	1.4	1.5	NA
EOA [cm^2^]	2.5	0.7	0.5	0.7	0.9	1.9	0.5	NA
Pressure recovery [mmHg]	4	11	13	16	16	4	17	NA
EOA for pressure recovery [cm^2^]	3.7	0.8	0.5	0.8	1.0	2.4	0.5	NA
ELCo [cm^2^]	3.7	0.8	0.5	0.8	1.0	2.4	0.5	NA
ELI [cm^2^/m^2^]	2.0	0.4	0.3	0.5	0.6	1.7	0.4	NA
Aortic sinus diameter (cm)	NA	2.3	2.9	2.9	3	2.4	1.7	2.6
Ascending aorta diameter (cm)	NA	4.3	4.3	3.9	4.7	2.5	4.2	4.3
LV EF [%]	NA	44	69	46.8	69.2	52.1	71	47
LV SV [mL]	NA	78.3	172.3	77.1	94.5	51.2	95	200
LV EDV [mL]	NA	178.1	249.5	164.8	136.5	98.2	133.8	424
LV ESV [mL]	NA	99.8	77.3	87.7	42.0	47.0	38.8	224
LV mass [g]	NA	274.4	249.7	91.4	183.3	42.0	189.9	179

NA, not available; TPG, transvalvular pressure gradient; BSA, body surface area; EOA, effective orifice area ELCo, energy loss coefficient, ELI, energy loss index LV, left ventricle; EF, ejection fraction; SV, stroke volume; EDV, end diastolic volume; ESV, end systolic volume.

* Total TKE was measured at the peak systole phase.

Note that TPG_max_ and TPG_mean_ are estimated based on the equation for the transvalvular pressure gradient (TPG = 4 × velcoity^2^).

TPG_max_ and TPG_mean_ are estimated based on the maximum and mean velocities of the valvular blood flow obtained from echo-Doppler measurements.

**Fig 5:**
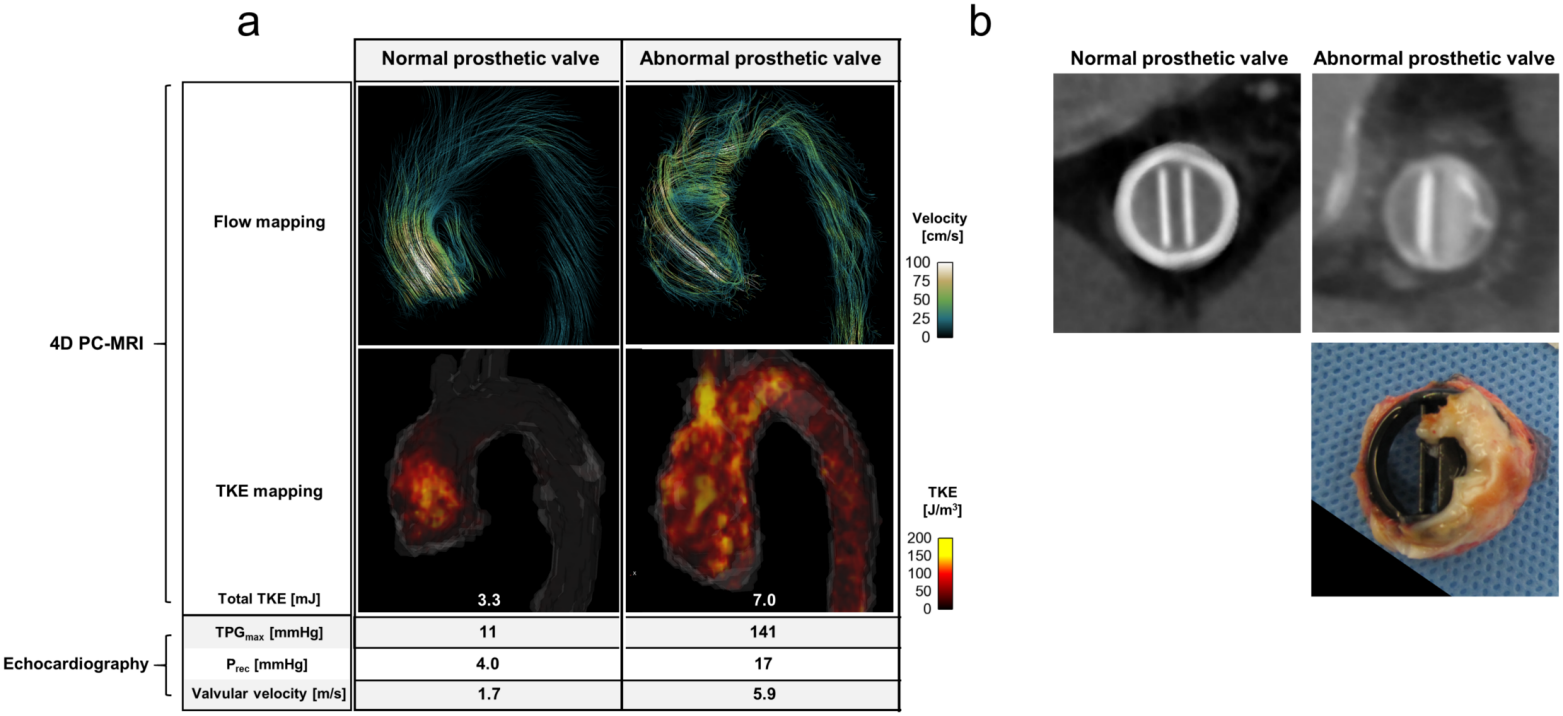
4D PC-MRI and echocardiographic findings in two patients with prosthetic valves. (a) Comparison of 4D PC-MRI derived velocity and TKE mapping with echocardiography parameters. (b) Comparison of normal and abnormal functioning prosthetic valves. Upper panel shows CT projection images of prosthetic valves. Lower panel shows an abnormal prosthetic valve removed from a patient. A substantial portion of the prosthetic valve is obstructed by pannus. Flow mapping indicates the flow streamlines. The uniform seed points within the thoracic aorta are used to visualize the streamlines. The TKE mapping is visualized by the volume rendering of the TKE within the entire thoracic aorta.

Two patients had prosthetic valves, one functioning normally and one abnormally. The normally functioning prosthetic valve generated non-helical blood flow through the ascending aorta, whereas the abnormal prosthetic valve generated a highly helical blood flow because one leaflet of the valve was not fully open due to pannus formation (Fig 5a and 5b). This case (#7) had a higher total TKE than did case #6 with the normal prosthetic valve; however, in spite of the extremely high TPG_max_ (141 mmHg), total TKE (7.0 mJ) was lower than those in patients with aortic stenosis with lower TPG_max_. Interestingly, blood flow in a patient with aortic regurgitation (case #8) induced a substantial total TKE (15.0 mJ) at systole, whereas TPG_max_ was small (22 mmHg; S3a Fig). In addition, the regurgitating blood flow through the partially unclosed aortic valve (S3b Fig) added 7.7 mJ to the total TKE at the ascending aorta during diastole.

## Discussion

The present study can be summarized as follows: measurement of TKE by using a clinical MRI scanner is feasible, and patients with aortic valve disease show high but variable values of TKE. TKE in the ascending aorta measured by 4D PC-MRI differs substantially from the TPG_max_ estimated by echocardiography because the increased stroke volume of the left ventricle, dilated ascending aorta, and regurgitating jet blood flow can be sources of turbulent flow without aortic valve stenosis, resulting in increased TKE in the ascending aorta compared with the healthy control. This implies that TKE quantification based on 4D PC-MRI measures phenomena that differ fundamentally from those measured by Doppler-based velocity and pressure parameters. TKE quantification reflects clinical variables such as the energy efficiency of blood flow and the cardiac work load. It may offer valuable information regarding the influence of valvular disease on the heart and systemic circulation. Therefore, TKE measurement by 4D PC-MRI can serve as an independent index that characterizes the amount of turbulence energy lost and can supplement the information obtained with conventional echocardiography.

Since the first development of a TKE measurement using PC-MRI, most *in vitro* demonstrations have been analyzed by computational fluid dynamics (CFD) [5, 7, 8]. However, a substantial discrepancy remains between CFD simulations and experimental measurements of stenosis blood flow because the transition from laminar to turbulent flow is still a challenging problem for CFD [19, 20]. In particular, a quantitative analysis of the transitional Re, velocity flow field, and the corresponding wall shear stress in the post-stenosis region is not yet available [14, 19–21]. Therefore, experimental verifications are required to confirm the TKE measurement with 4D PC-MRI.

For the laminar-flow regime (Re < 400), the results of the TKE measurement using 4D PC-MRI deviate strongly from the PIV results. While blood flow through the stenosis (50% reduction in diameter) becomes turbulent for Re > 400 [14], the flow for Re < 400 is not fully turbulent; however, intermittent velocity fluctuations still appear in the post-stenosis region [22]. Because PIV continuously measures flow velocity during a relatively long time (a few seconds to minutes), it can account for the intermittent velocity fluctuations; therefore, a low TKE was measured at Re = 337 (Fig 1b). However, the 4D PC-MRI measurement detects almost no TKE for Re < 400 because k-space data filling and IVSD-based turbulence quantification of 4D PC-MRI do not correctly reflect the intermittent variation in the velocity. As a result, the TKE measurements by PIV and PC-MRI for the laminar-flow region (Re < 400) do not show a strong linear correlation (R^2^ < 0.341). This result also implies that 4D PC-MRI may not accurately describe the TKE distribution at low Re flow (e.g., diastolic blood flow).

The present study also assesses the feasibility of TKE measurements *in vivo* for patients with valvular conditions such as aortic stenosis, prosthetic valves, and aortic regurgitation. This analysis reveals a discrepancy between the total TKE and TPG_max_. Although patients with aortic stenosis had a similar range of transvalvular velocity (4.2–4.7 m/s) and TPG_max_ (70–89 mmHg), their TKE distribution varied widely because the development of turbulence is influenced by the geometry of the aortic valve, the size of the ascending aorta, and the corresponding blood-flow patterns. Elevation of total TKE in the patient with an abnormal prosthetic valve was not as high as in the patients with aortic stenosis, although transvalvular velocity and TPG_max_ in this case were extremely high (5.9 m/s and 141 mmHg, respectively). Conversely, although the patient with aortic regurgitation had only modest TPG_max_ (22 mmHg), a substantial amount of TKE in the ascending aorta was measured during both systole and diastole. This result indicates that the increased stroke volume of the left ventricle, dilated ascending aorta, and regurgitating jet blood flow can be sources of turbulent flow without aortic valve stenosis, resulting in increased TKE in the ascending aorta compared with the healthy control patient. This also indicates that TKE quantification elucidates aspects of the hemodynamics that differ from those characterized by echo-Doppler parameters.

Turbulent flow usually occurs when blood passes through pathological heart valves such as aortic valve stenosis, bicuspid aortic valves, and prosthetic aortic valves [23, 24]. The TKE distribution is closely related to the fluid-energy loss and the pressure drop through the heart valve. Therefore, the increase in the TKE is also closely related to an increased work load for the myocardium. One of the standard methods for estimating the pressure drop through the heart valve in clinical practice is to measure the peak velocity in the valve region and apply a simplified Bernoulli equation. Although this method is preferred because of its versatility and noninvasiveness, it often overestimates the pressure drop because it does not consider pressure recovery in the post-valve region [2]. Therefore, previous studies have attempted to compensate for pressure recovery by adding the diameter of the post-valve region as a weighting coefficient [2]; however, this indirect estimate of turbulence is limited because the amount of turbulence energy varies with the hemodynamic parameters (e.g., flow angle, heart pulsatility, blood viscosity, vessel geometry). In the present study, *in vivo* testing revealed a substantial discrepancy between total TKE and TPG_max_, in contrast to the *in vitro* phantom studies, which showed that the total TKE and TPG_max_ had a strong linear correlation (R^2^ = 0.985; Fig 2). This implies that, even though TPG_max_ and TKE are associated in the ideal stenotic flow [2, 25], various clinical parameters, including the eccentricity of the jet flow at the heart valve and the size of the aorta, can influence the development of turbulent flow and the true pressure gradient. A previous study also reported that the relationship between TKE and pressure gradient can vary depending on the configuration of the stenosis, such as a post-stenosis dilatation [8]. This also implies that the stenosis phantom can provide a different relationship between TKE and pressure gradient (e.g., the regression slope) depending on the post-stenotic dilation of the stenosis phantom.

Alternatively, we consider that TKE quantification based on 4D PC-MRI measures phenomena that differ fundamentally from those characterized by Doppler-based velocity and pressure parameters. TKE quantification reflects clinical variables such as the energy efficiency of blood flow and the cardiac work load. It may offer valuable information on how valvular disease affects the heart and systemic circulation. Therefore, TKE measurement by 4D PC-MRI can serve as an independent index that characterizes the amount of turbulence energy lost and can supplement information obtained by using conventional echocardiography.

Note that the segmentation of the aorta can influence the region and total TKE distribution. In the present study, the aorta is manually segmented, so an accurate segmentation of the aortic-valve regions was not feasible based on the magnitude and phase data because those data from 4D PC-MRI have low resolution and poor contrast near the aortic valve. Therefore, in the present study, we exclude the TKE near the aortic valve. In addition, the TKE near the prosthetic valve is excluded because it induces significant signal loss near the aortic valve level. Note also that signal loss due to the prosthetic valve can lead to an underestimate of the total TKE in patients with such a valve compared with other patients.

In addition, note that PIV in the present study is used as a reference standard but not a gold standard. As described in the methods section, PIV also contains errors of around ±3% and up to ±40% depending on the measurement location. Therefore, in the present study, differences in TKE obtained using PIV and that using 4D PC-MRI can be attributed to inaccuracies in both 4D PC-MRI and PIV.

A limitation of the present study is that the *in vitro* verification used the constant-flow conditions Re < 945. Because the flow field is influenced by the acceleration and deceleration of the flow, the pulsatility of the flow can influence the velocity field and the pressure [26]. Therefore, the correlation between pressure and TKE obtained from the present study can vary under pulsatile flow conditions, so further study is required to clarify the relationship between pressure and TKE under pulsatile flow. Considering that Re in the ascending aorta reaches up to 5000 or 10 000 [24], the relationship between TKE and pressure drop at higher Re remains to be investigated. In addition, because this study uses an idealized stenosis shape in the phantom, TKE measurements in blood flow through various naturally shaped aortic valves and stenoses are also required. Finally, the number of cases in the *in vivo* study was insufficient to permit a statistical analysis.

In conclusion, the results of the present study suggest that TKE measurement using MRI may prove beneficial as an energy-loss index for characterizing blood flow through the aortic valve. However, further clinical studies are necessary to draw more definitive conclusions.

## Supporting Information

S1 Fig Schematic drawings of experimental setup. (a) Flow circuit system for MRI and PIV measurements. (b) Experimental setup for PIV measurement.
(TIF)

S2 Fig Schematic of velocity and turbulence quantification using 4D-MRI. (TIF)

S3 Fig 4D PC-MRI and echocardiographic finding in patients with aortic regurgitation. (a) Comparison of 4D PC-MRI-derived velocity and TKE mapping with echocardiography parameters. (b) CT images of the aortic valve at systole (upper right), diastole (lower right), and 3D reconstruction image of the aorta (left). Flow mapping indicates the flow streamlines. The uniform seed points within the thoracic aorta are used to visualize the streamlines.
(TIF)

S1 Table Imaging and flow parameters for *in vitro* phantom studies. (DOCX)

S2 Table Imaging and flow parameters for *in vivo* patient studies. (DOCX)

S3 Table Linear regression and Bland–Altman parameters for *in vitro* phantom studies. (DOCX)

## Abbreviations

4D PC-MRI: four-dimensional phase-contrast magnetic resonance imaging
MRI: magnetic resonance imaging
IVSD: intravoxel standard deviation
TKE: turbulence kinetic energy
Re: Reynolds number
VENC: velocity encoding parameters
iPAT: integrated parallel acquisition technique
PIV: particle image velocimetry
TE: Echo time
TPG: transvalvular pressure gradient
