# Supplementary figures and images for: Turbulent Kinetic Energy Measurement Using Phase Contrast MRI for Estimating the Post-Stenotic Pressure Drop: In Vitro Validation and Clinical Application
Source: PLoS One. 2016 Mar 15;11(3):e0151540. doi: 10.1371/journal.pone.0151540 (PMC4792455; doi:10.1371/journal.pone.0151540)

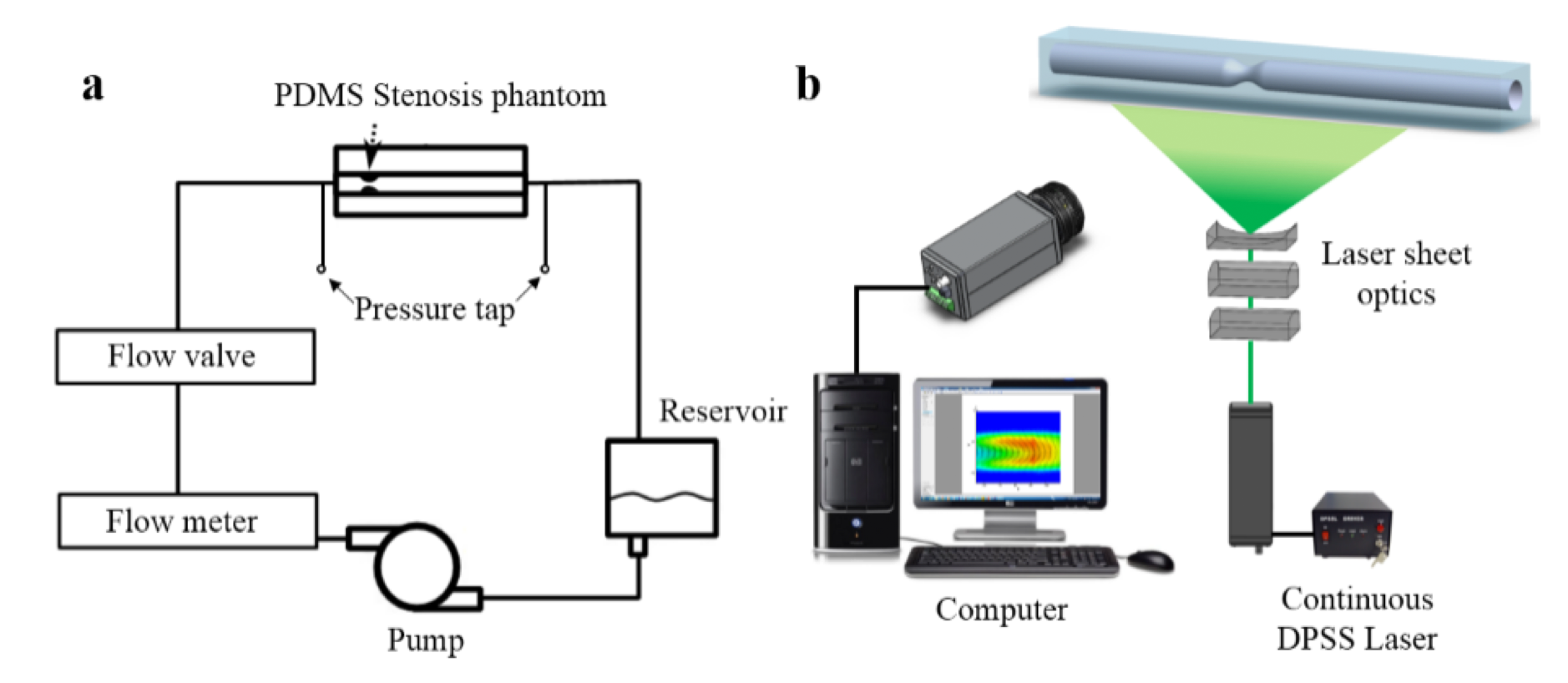

Supplement: S1 Fig — (a) Flow circuit system for MRI and PIV measurements. (b) Experimental setup for PIV measurement. (TIF) [file pone.0151540.s001.tif]

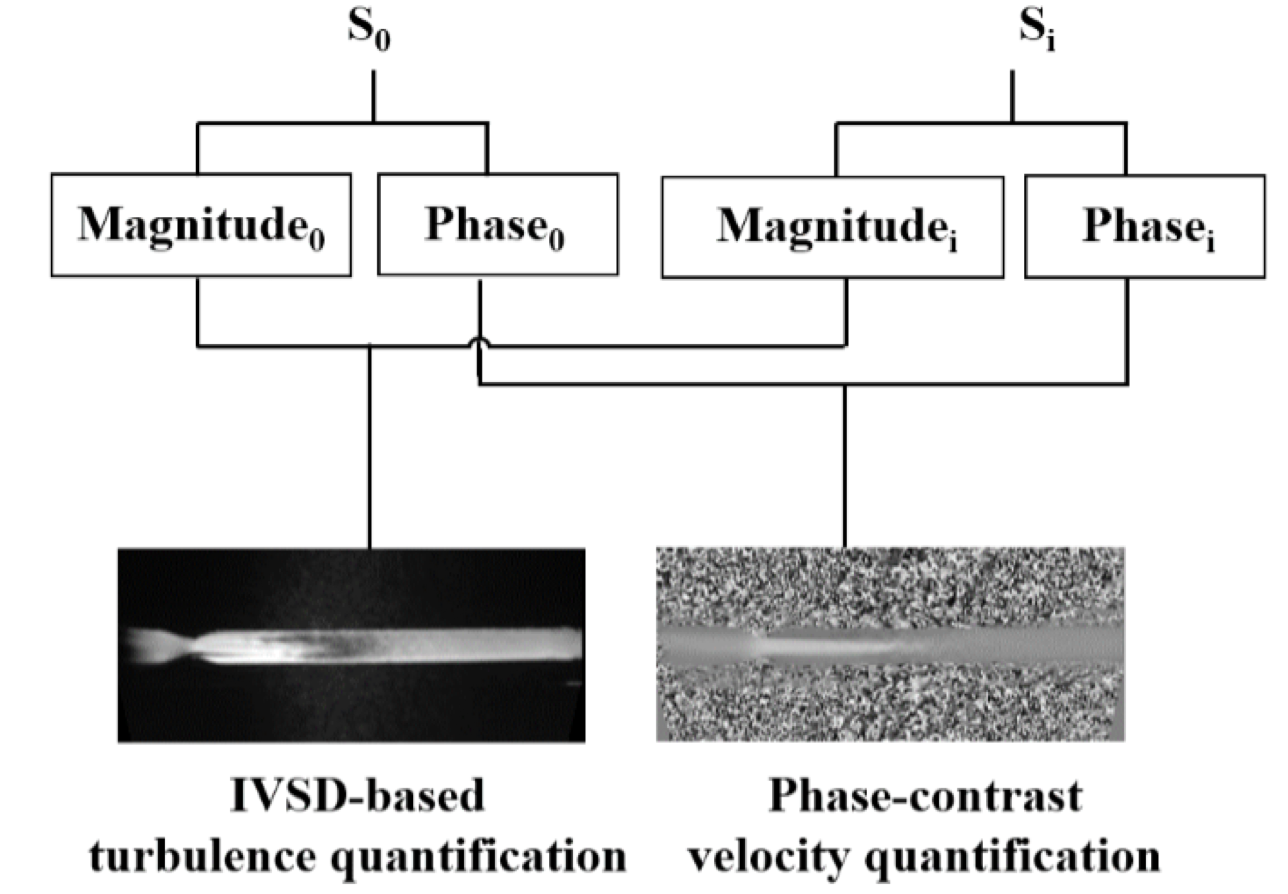

Supplement: S2 Fig — (TIF) [file pone.0151540.s002.tif]

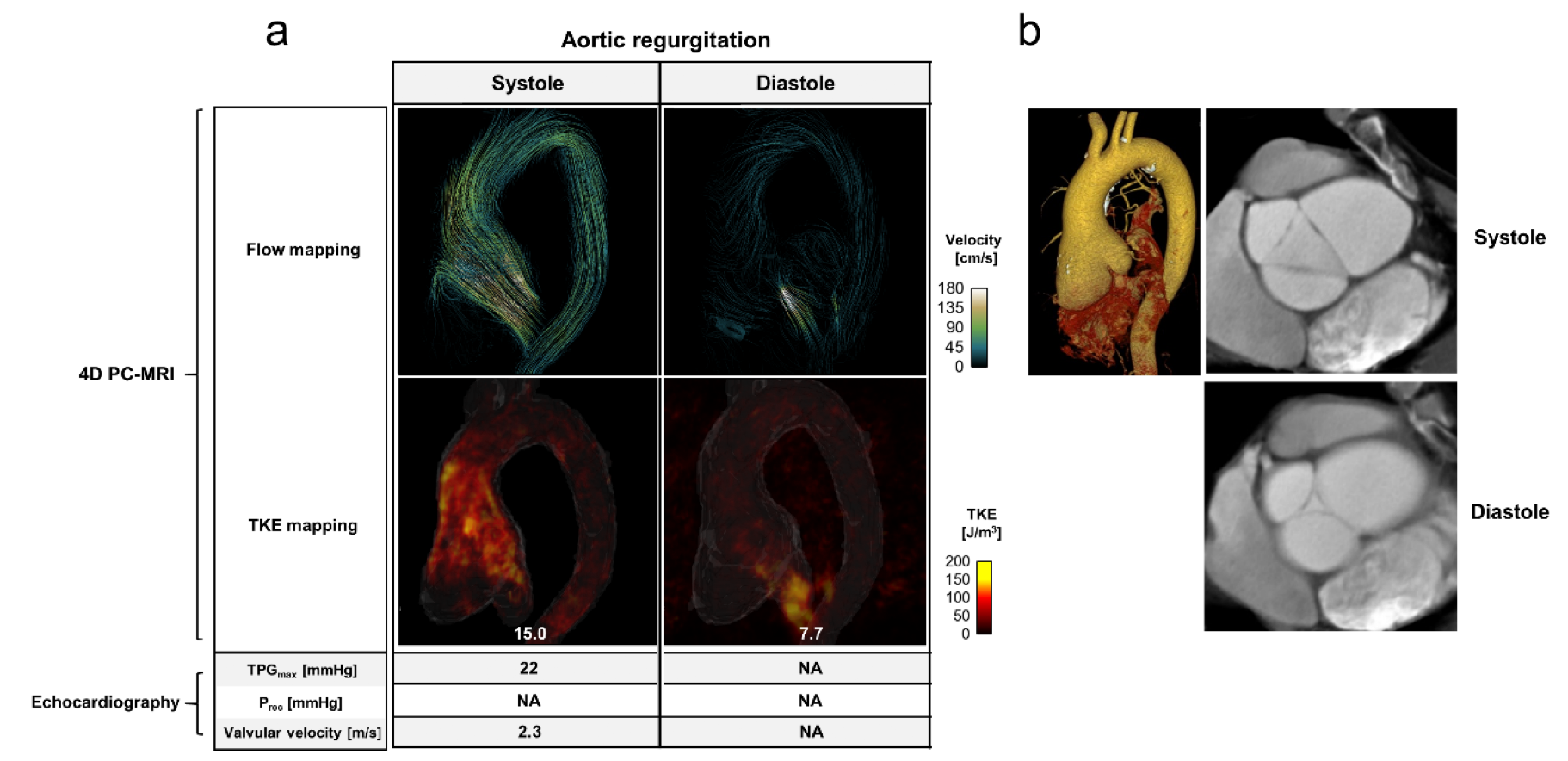

Supplement: S3 Fig — (a) Comparison of 4D PC-MRI-derived velocity and TKE mapping with echocardiography parameters. (b) CT images of the aortic valve at systole (upper right), diastole (lower right), and 3D reconstruction image of the aorta (left). Flow mapping indicates the flow streamlines. The uniform seed points within the thoracic aorta are used to visualize the streamlines. (TIF) [file pone.0151540.s003.tif]
