# Supplementary material for: Turbulent Kinetic Energy Measurement Using Phase Contrast MRI for Estimating the Post-Stenotic Pressure Drop: In Vitro Validation and Clinical Application
Source: PLoS One. 2016 Mar 15;11(3):e0151540. doi: 10.1371/journal.pone.0151540 (PMC4792455; doi:10.1371/journal.pone.0151540)
Supplement: S1 Table — (DOCX) [file pone.0151540.s004.docx]

**S1 Table.** Imaging and flow parameters for *in vitro* phantom studies.

| **Flow Rate**  **[liter/min]** | **Re** | **Velocity measurement** | **Turbulence measurement** | **TE**  **[ms]** | **Temporal resolution**  **[ms]** | **Voxel size  [mm × mm × mm]** | **Matrix size  [pixel × pixel × pixel]** | **Flip angle [degree]** | **Total Scan time [min]** |
| --- | --- | --- | --- | --- | --- | --- | --- | --- | --- |
|  |  |  |  |  |  |  |  |  |  |
|  |  | **VENC**  **[cm/s]** | **VENC**  **[cm/s]** |  |  |  |  |  |  |
|  |  |  |  |  |  |  |  |  |  |
|  |  |  |  |  |  |  |  |  |  |
| 8.7 | 945 | 260 | 78 | 6.76 | 92.72 | 1.4 × 1.4 × 1.4 | 256 × 256 × 6 | 10° | 4 |
| 8.2 | 890 | 260 | 78 |  |  |  |  |  |  |
| 7.6 | 825 | 230 | 69 |  |  |  |  |  |  |
| 7 | 760 | 200 | 60 |  |  |  |  |  |  |
| 6.5 | 706 | 200 | 60 |  |  |  |  |  |  |
| 5.4 | 586 | 170 | 51 |  |  |  |  |  |  |
| 4.9 | 532 | 145 | 44 |  |  |  |  |  |  |
| 4.2 | 456 | 115 | 35 |  |  |  |  |  |  |
| 3.6 | 391 | 90 | 27 |  |  |  |  |  |  |
| 3.1 | 337 | 90 | 27 |  |  |  |  |  |  |
| 2.4 | 261 | 75 | 22 |  |  |  |  |  |  |
| 1.3 | 141 | 50 | 15 |  |  |  |  |  |  |

Re, Reynolds number; TE, echo time; VENC, velocity encoding.
