# Supplementary material for: Turbulent Kinetic Energy Measurement Using Phase Contrast MRI for Estimating the Post-Stenotic Pressure Drop: In Vitro Validation and Clinical Application
Source: PLoS One. 2016 Mar 15;11(3):e0151540. doi: 10.1371/journal.pone.0151540 (PMC4792455; doi:10.1371/journal.pone.0151540)
Supplement: S2 Table — (DOCX) [file pone.0151540.s005.docx]

**S2 TABLE.** Imaging and flow parameters for *in vivo* patient studies.

| **Case** | **Study group** | **Velocity measurement** | | | **TKE measurement** | | | **Matrix size  [pixel × pixel × pixel]** | **Scan time [min]** | **Voxel size  [mm × mm × mm]** | **Flip angle [degree]** |
| --- | --- | --- | --- | --- | --- | --- | --- | --- | --- | --- | --- |
|  |  | **VENC [cm/s]** | **TE* [ms]** | **Temporal resolution [ms]** | **VENC [cm/s]** | **TE* [ms]** | **Temporal resolution [ms]** |  |  |  |  |
|  |  |  |  |  |  |  |  |  |  |  |  |
|  |  |  |  |  |  |  |  |  |  |  |  |
| 1 | Normal | 250 | 4.64 | 36.72 | 50 | 5.4 | 39.6 | 132×192×30 | 29 | 2×2×2 | 10° |
| 2 | Aortic stenosis (TAV) | 550 | 4.28 | 35.28 | 180 | 4.48 | 36.08 | 132×192×48 | 53 |  |  |
| 3 | Aortic stenosis (TAV),  Aortic regurgitation | 400 | 4.43 | 35.88 | 133 | 4.43 | 35.88 | 132×192×42 | 43 |  |  |
|  |  |  |  |  |  |  |  |  |  |  |  |
| 4 | Aortic stenosis (BAV) | 360 | 4.28 | 35.28 | 120 | 4.7 | 36.96 | 132×192×44 | 42 |  |  |
| 5 | Aortic stenosis (TAV) | 300 | 4.28 | 35.28 | 100 | 4.84 | 37.52 | 132×192×44 | 66 |  |  |
| 6 | Normal prosthetic valve | 210 | 4.36 | 35.6 | 70 | 5.12 | 38.64 | 132×192×40 | 54 |  |  |
| 7 | Abnormal prosthetic valve | 280 | 4.28 | 35.28 | 93 | 4.87 | 37.64 | 132×192×44 | 35 |  |  |
| 8 | Aortic regurgitation | 250 | 4.28 | 35.28 | 85 | 4.95 | 37.96 | 132×192×48 | 63 |  |  |

*TE, echo time; VENC, velocity encoding; TKE, turbulent kinetic energy; TAV, tricuspid aortic valve; BAV, bicuspid aortic.
