# Supplementary material for: Turbulent Kinetic Energy Measurement Using Phase Contrast MRI for Estimating the Post-Stenotic Pressure Drop: In Vitro Validation and Clinical Application
Source: PLoS One. 2016 Mar 15;11(3):e0151540. doi: 10.1371/journal.pone.0151540 (PMC4792455; doi:10.1371/journal.pone.0151540)
Supplement: S3 Table — (DOCX) [file pone.0151540.s006.docx]

**S3 TABLE.** Linear regression and Bland–Altman parameters for *in vitro* phantom studies.

| **Flow characteristics** | **Q  [liter/min]** |  | **V_MRI_ = a*V_PIV_ + b** | | | | **V_PIV-_V_MRI_ [cm/s] (Mean±1.96SD)** | **TKE_MRI_ = a*TKE_PIV_ + b** | | | | **TKE_PIV-_TKE_MRI_ [J/m^3^] (Mean±1.96SD)** |
| --- | --- | --- | --- | --- | --- | --- | --- | --- | --- | --- | --- | --- |
|  |  | **Re** | **a** | **b** | **SEE** | **R^2^** |  | **a** | **b** | **SEE** | **R^2^** |  |
| Turbulent flow | 8.7 | 945 | 1.06 | -6.03 | 12.84 | 0.885 | 3.66±25.47 | 1.13 | 8.55 | 33.29 | 0.738 | -16.75±25.47 |
|  | 8.2 | 890 | 1.09 | -5.75 | 10.96 | 0.909 | 2.34±22.22 | 1.08 | 12.04 | 30.81 | 0.735 | -16.60±60.78 |
|  | 7.6 | 825 | 1.07 | -6.40 | 11.00 | 0.896 | 3.79±21.98 | 0.99 | 14.47 | 29.00 | 0.703 | -14.20±56.85 |
|  | 7 | 760 | 1.10 | -5.37 | 10.44 | 0.896 | 2.05±21.19 | 0.91 | 15.45 | 28.95 | 0.640 | -10.80±57.18 |
|  | 6.5 | 706 | 1.10 | -6.57 | 10.85 | 0.877 | 3.43±21.91 | 0.82 | 16.74 | 24.02 | 0.646 | -8.20±48.97 |
|  | 5.4 | 586 | 1.08 | -5.28 | 9.14 | 0.880 | 3.07±18.29 | 0.81 | 16.21 | 21.77 | 0.629 | -8.14±44.54 |
|  | 4.9 | 532 | 0.85 | 1.88 | 11.47 | 0.700 | 1.81±23.27 | 0.89 | 13.85 | 17.22 | 0.729 | -9.64±34.38 |
|  | 4.2 | 456 | 1.06 | -3.14 | 7.03 | 0.890 | 1.89±13.94 | 0.73 | 14.07 | 13.95 | 0.666 | -4.69±30.78 |
| Laminar flow | 3.6 | 391 | 0.98 | -2.33 | 6.41 | 0.874 | 2.67±12.57 | 0.42 | 12.52 | 13.14 | 0.341 | 5.34±36.15 |
|  | 3.1 | 337 | 1.00 | -2.26 | 6.34 | 0.839 | 2.26±12.43 | -0.02 | 15.23 | 11.08 | 0.002 | 9.41±39.31 |
|  | 2.4 | 261 | 0.91 | -1.73 | 4.72 | 0.842 | 2.99±9.47 | -0.10 | 7.57 | 7.90 | 0.004 | 2.32±18.99 |
|  | 1.3 | 141 | 0.90 | -0.21 | 1.15 | 0.964 | 1.01±2.62 | 0.28 | 1.54 | 3.75 | 0.183 | -1.07±7.37 |

Re, Reynolds number; Q, flow rate; V, velocity; TKE, turbulent kinetic energy; SD, standard deviation, SEE, standard error of the estimate.
